# Impact of Smoluchowski Temperature and Maxwell Velocity Slip Conditions on Axisymmetric Rotated Flow of Hybrid Nanofluid past a Porous Moving Rotating Disk

**DOI:** 10.3390/nano12020276

**Published:** 2022-01-15

**Authors:** Umair Khan, Aurang Zaib, Iskandar Waini, Anuar Ishak, El-Sayed M. Sherif, Wei-Feng Xia, Noor Muhammad

**Affiliations:** 1 Department of Mathematical Sciences, Faculty of Science and Technology, Universiti Kebangsaan Malaysia UKM, Bangi 43600, Selangor, Malaysia; umairkhan@iba-suk.edu.pk (U.K.); anuar_mi@ukm.edu.my (A.I.); 2 Department of Mathematics and Social Sciences, Sukkur IBA University, Sukkur 65200, Pakistan; 3 Department of Mathematical Sciences, Federal Urdu University of Arts, Science & Technology, Gulshan-e-Iqbal Karachi 75300, Pakistan; aurangzaib@fuuast.edu.pk; 4 Fakulti Teknologi Kejuruteraan Mekanikal dan Pembuatan, Universiti Teknikal Malaysia Melaka, Durian Tunggal 76100, Melaka, Malaysia; iskandarwaini@utem.edu.my; 5 Mechanical Engineering Department, College of Engineering, King Saud University, Riyadh 11423, Saudi Arabia; esherif@ksu.edu.sa; 6 School of Engineering, Huzhou University, Huzhou 313000, China; 7 Abdus Salam School of Mathematical Sciences, Government College University, Lahore 54600, Pakistan; noor@math.qau.edu.pk; 8 Department of Mathematics, Texas A&M University, College Station, TX 77840, USA

**Keywords:** hybrid nanofluid, axisymmetric rotating flow, Maxwell velocity slip, Smoluchowski temperature slip, rotating disk

## Abstract

Colloidal suspensions of regular fluids and nanoparticles are known as nanofluids. They have a variety of applications in the medical field, including cell separation, drug targeting, destruction of tumor tissue, and so on. On the other hand, the dispersion of multiple nanoparticles into a regular fluid is referred to as a hybrid nanofluid. It has a variety of innovative applications such as microfluidics, heat dissipation, dynamic sealing, damping, and so on. Because of these numerous applications of nanofluids in minds, therefore, the objective of the current exploration divulged the axisymmetric radiative flow and heat transfer induced by hybrid nanofluid impinging on a porous stretchable/shrinkable rotating disc. In addition, the impact of Smoluchowski temperature and Maxwell velocity slip boundary conditions are also invoked. The hybrid nanofluid was formed by mixing the copper (Cu) and alumina (Al_2_O_3_) nanoparticles scattered in the regular (viscous) base fluid (H_2_O). Similarity variables are used to procure the similarity equations, and the numerical outcomes are achieved using bvp4c in MATLAB software. According to the findings, double solutions are feasible for stretching (λ>0) and shrinking cases (λ<0). The heat transfer rate is accelerated as the hybrid nanoparticles increases. The suction parameter enhances the friction factors as well as heat transfer rate. Moreover, the friction factor in the radial direction and heat transfer enrich for the first solution and moderate for the second outcome due to the augmentation $\delta_{1}$, while the trend of the friction factor in the radial direction is changed only in the case of stretching for both branches.

## 1. Introduction

The study of nanofluids piqued the interest of researchers because of different applications in science and technology, like hybrid-powered engines, fuel cells, measures of different heat transport with pharmaceutical, and micro-electronic dealings. Nanofluids are composed of nanoparticles, and the presence of ultrafine nanoparticles magnifies thermal conductivity. Choi and Eastman [1] first proposed the idea of nanofluids by dispersion of fluids comprising ultra-fine particles. Later, several researchers investigated the mechanism of nanofluid in different geometrical domains: such as Khan and Pop [2] addressed the flow problem past a stretchable sheet containing nanofluids. The effect of free buoyancy flow via a vertical plate induced by nanofluid saturated in a porous medium was inspected by Gorla and Chamkha [3]. Xu et al. [4] presented an exact solution of the time-dependent flow induced by thin fluid film subject to the stretchable sheet. Gireesha et al. [5] examined the impact of dust particles scattered in a nanofluid flow from a stretched surface. Krishnamurthy et al. [6] scattered fluid particles in a magnetic fluid flow induced by nanofluid past an exponentially stretchable sheet with viscous dissipation. Makarem et al. [7] numerically inspected the fluid flow and features of heat transfer with distinct nanofluids on a stretchable surface. Ghalambaz et al. [8] discussed the features of fluid flow and analysis of heat transfer through a vertical plate saturated in a porous media packed with nano-encapsulated suspensions. They observed that the heat transfer uplifts in the presence of nano-encapsulated materials. Wakif and Sehaqui [9] revised the two-phase model by utilizing the water-based nanofluid to investigate the features of metal nanoparticles with a magnetic effect. Hajjar et al. [10] inspected the free convective flow of time-periodic with heat transfer scattered nano-encapsulated materials in a cavity and utilized the finite element technique to get a solution. The impact of nanofluid on gravity-driven flow induced by thixotropic fluid comprising microorganisms through a vertical sheet was examined by Koriko et al. [11]. Gul et al. [12] scrutinized the influence of thermophoresis and Brownian motion on thin fluid film induced by Carreau fluid from a stretched sheet with couple stress. The impact of magnetic field on the unsteady thin film flow of nanofluid with irregular convective stream past an inclined stretched surface is inspected by Saeed et al. [13]. Mathew et al. [14] investigated the effects of nanoparticle shape and slips on the magneto stagnation-point flow induced by nanofluid with chemical reaction and thermal radiation. Gul et al. [15] investigated a 3D MHD steady flow of Casson nanofluid induced by gyrotactic microorganisms through the gap of a cone and disk and developed the homotopy method to find the result. Recently, Gul et al. [16] examined the dissipative flow of nanofluid past a time-dependent turning disc with the magnetic field. They observed that the magnetic factor declines the velocities in radial and transverse directions.

Generally, each nanofluid possesses only one nanoparticle, whereas the hybrid nanofluid consists of two different nanoparticles dispersed in a different element regular fluid or a combination of component regular fluids. Hybrid nanofluids are created by combining two distinct nanomaterials to improve thermal and rheological properties. The main objective of hybrid nanofluid advancement is to effectively manage heat transport phenomenon in the examination of flow characteristics field. It has a wide range of technological applications including microfluidics, damping, acoustics, naval, heat dissipation, dynamic sealing, and so on. Suresh et al. [17] investigated the effect of dissipation on the time-dependent free buoyancy consequences of a hybrid nanofluid flow through a circular tube. The analysis of mixed convection flow induced by hybrid nanofluid was experimentally inspected by Momin [18]. Takabi and Salehi [19] scrutinized the hybrid nanofluid within a sinusoidal enclosure. Devi and Devi [20] presented the numerical solution of hybrid liquids past stretchable surfaces. They discovered an enrich the heat transport, which was followed by an increase in nanoparticle concentration. Hayat and Nadeem [21] described an escalation in heat transfer provided by an Ag-CuO/water nano solution. Rostami et al. [22] investigated the mixed convective flow of a water-based silica-alumina hybrid nanofluid in conjunction with a vertical plate by imposing linearly unstable temperatures with analysis of heat transfer. Double solutions were presented for opposing flow as well as for assisting flow. A parallel problem was surveyed by Zainal et al. [23] by considering convective boundary conditions along with different nanomaterials. Acharya et al. [24] discussed the characteristics of Hall current through a moving disk by including the Cu/TiO_2_nanomaterials suspended into base fluid water along with subject to thermal radiation and magnetic effects. Recently, several researchers [25,26,27,28,29,30,31,32,33,34] explored the significance of hybrid nanofluid flow with different aspects.

Over the last decade or so, there was a lot of interest in the analysis of stagnation point flow past a rotating disc. Hiemenz [35] discovered an analytic solution to N-S equations describing 2D steady flow handled orthogonally by an extended flat plate. Later the aforesaid problem was extended by Homann [36] to include an axisymmetric flow case. Hannah [37] used the irrotational far-field flow to extend Homann’s [36] classic stagnation-point flow on a flat surface to survey the flow dynamics versus a spinning disc. Alternatively, Agrawal [38] formed a novel axially symmetric stagnation point flow on rotating external flow and normal impact to an infinite or inestimable or vary far plane wall. Then, Weidman [39] prolonged Agrawal’s problem numerically. The impact of magnetic field on a 3D rotated flow towards a stagnation-point through a stretched radial rotating disk was inspected by Turkyilmazoglu [40]. Weidman [41,42] scrutinized the Agrawal axisymmetric flow near a stagnation-point over a flat sheet and stretched sheet, respectively. Recently, Waini et al. [43] reconnoitered the Agrawal’s flow and the features of heat transfer along with hybrid nanoparticles from a stretchable/shrinkable disc.

The component of tangential velocity at the surface is proportional to the shear stress wall is known as the slip boundary condition. Navier [44] coined the term slip boundary condition after discovering a relationship between shear stress and slip rate. Maxwell [45] proposed the simplest explanation of the phenomena of velocity slip, which depends on a gradient of velocity exerting normal to a creep term and the surface. Smoluchowski [46] later introduces the idea of temperature slip. Ramya et al. [47] investigated the effect of thermal and velocity slips on fluid dispersed in a nanofluid. Recently, Khashi et al. [48] scrutinized the impacts of convective boundary stipulations and slip on a 3D flow of a hybrid nanofluid past a stretchable/shrinkable sheet.

The goal of the current study is to look into axisymmetric rotating radiative flow and heat transfer induced by hybrid nanofluid through a stretchable/shrinkable sheet with Smoluchowski temperature and Maxwell velocity slip. The following are the key points of research of the current study:

- The impacts of Smoluchowski temperature and Maxwell velocity slip on the axisymmetric rotating flow were not explored yet.
- The radiation effect on the axisymmetric rotating flow induced by hybrid nanofluid was not yet considered.
- Double solutions of axisymmetric rotating flow past a moving rotating disk in the presence of hybrid nanofluid were not presented before.

## 2. Mathematical Formulation

**Figure 1:**
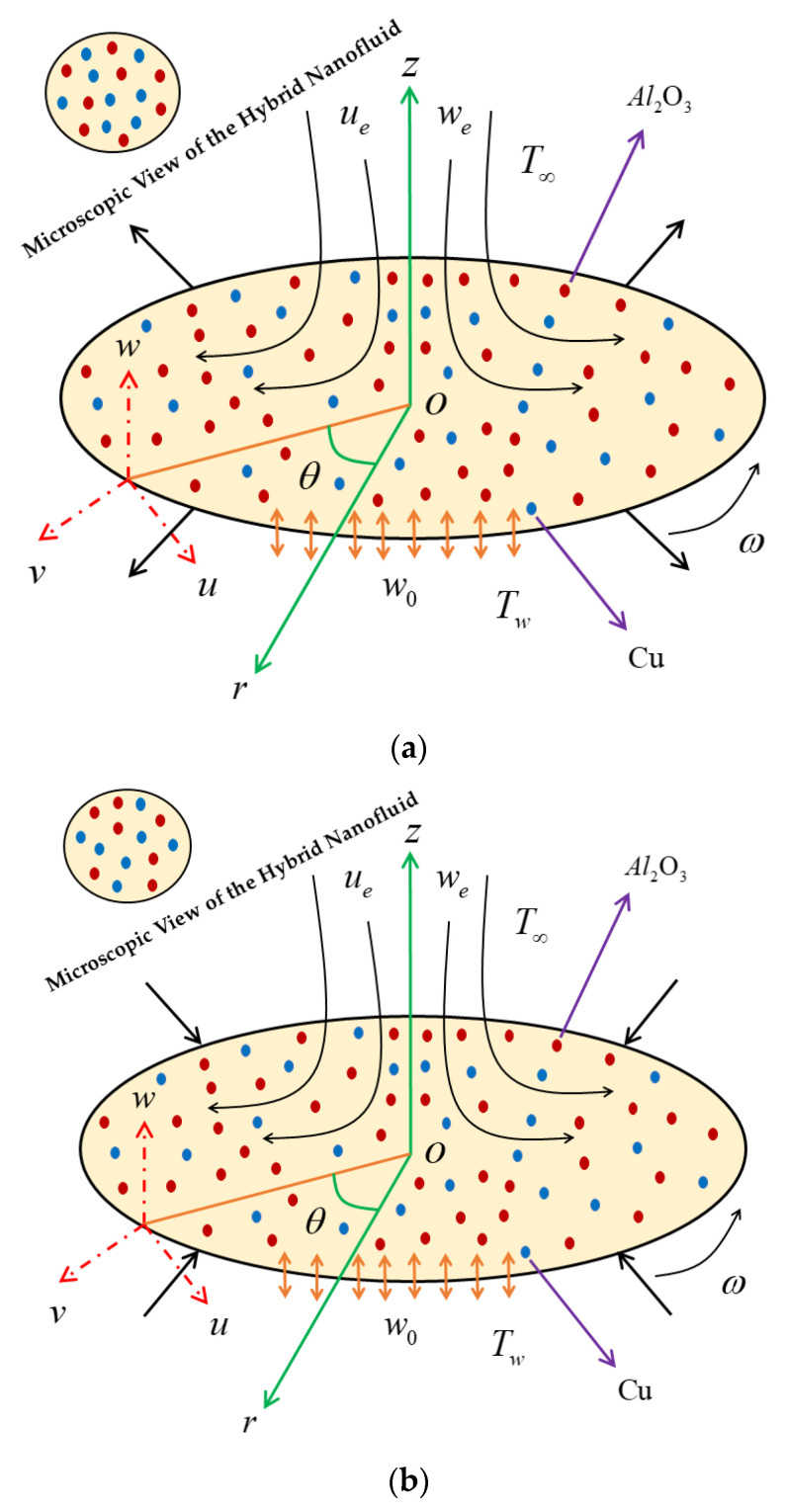
Physical model of problem. (**a**) Stretching disk, (**b**) Shrinking disk.

The configuration of the Agrawal flow problem is schematically shown in Figure 1, where the steady radiative axisymmetric flow of heat transfer along with rotational stagnation-point induced by hybrid nanoparticles impinging radially a porous shrinkable/stretchable rotating disk is contemplated. The problem is initially expressed in terms of cylindrical coordinates (z,r,θ) considered in the following axial, radial, and azimuthal directions, respectively, with the associated component of velocities (w,u,v). The Agrawal flow is symmetric to the rθ-plane and also axisymmetric about the axial direction (z-axis), i.e., the variation along the coordinate θ (azimuthal direction) is ignored. The stagnation line is located at z=0 and the region of the flow dynamics is in the upper half-plane. Therefore, the stretching/shrinking disk is rotating about the axial direction (z-axis) together with a fixed angular velocity ω. In addition, the hybrid nanofluid is composed of two dissimilar nanoparticles (i.e., alumina (Al_2_O_3_) and copper (Cu)) along with viscous pure fluid (water). The physical properties of the binary hybrid nanomaterials are taken to be in equilibrium and no-slip occurs between them. It is assumed that the component of the free stream velocities is characterized by $u_{e}\left(r,z\right)=2arz$, $v_{e}\left(r,z\right)=0$ and $w_{e}\left(r,z\right)=-2az^{2}$, where a is a constant parameter measuring the strength of the Agrawal flow having units ${\left(\mathrm{LT}\right)}^{-1}$, see Weidman [39]. Moreover, the surface velocity at the wall z=0 is denoted by $u_{w}=a^{2/3}\upsilon_{f}^{1/3}r$ along with Maxwell slip velocity [45] having slip length $\frac{2-\sigma_{v}}{\sigma_{v}}\lambda_{0}\frac{\partial u}{\partial z}$ were implemented to study the velocity slip effect and further $v_{w}=r\omega$, here, ω is the unchanging rotational speed of the disc, however, the uniform mass flux velocity via the wall is equal to $w_{0}$, where $w_{0}<0$ for suction and $w_{0}>0$ for blowing. The constant wall surface temperature $T_{w}$ along with the Smoluchowski slip temperature [46] is indicated by $\frac{2-\sigma_{T}}{\sigma_{T}}\left(\frac{2\gamma }{\gamma +1}\right)\frac{\lambda_{0}}{\mathrm{Pr}}\frac{\partial T}{\partial z}$, whereas the constant ambient temperature is signified by $T_{\infty }$ with the case $T_{w}>T_{\infty }$. Under the impact of the aforementioned assumptions, the modeled governing equations in form of PDEs are premeditated by (see Weidman [39]):

(1) $$\frac{\partial u}{\partial r}+\frac{u}{r}+\frac{\partial w}{\partial z}=0,$$

(2) $$u\frac{\partial u}{\partial r}-\frac{v^{2}}{r}+w\frac{\partial u}{\partial z}=-\frac{1}{\rho_{hnf}}\frac{\partial P}{\partial r}+\frac{\mu_{hnf}}{\rho_{hnf}}\left(\frac{\partial^{2}u}{\partial r^{2}}+\frac{1}{r}\frac{\partial u}{\partial r}-\frac{u}{r^{2}}+\frac{\partial^{2}u}{\partial z^{2}}\right),$$

(3) $$u\frac{\partial v}{\partial r}+\frac{uv}{r}+w\frac{\partial v}{\partial z}=\frac{\mu_{hnf}}{\rho_{hnf}}\left(\frac{\partial^{2}v}{\partial r^{2}}+\frac{\partial }{\partial r}\left(\frac{v}{r}\right)+\frac{\partial^{2}v}{\partial z^{2}}\right),$$

(4) $$u\frac{\partial w}{\partial r}+w\frac{\partial w}{\partial z}=-\frac{1}{\rho_{hnf}}\frac{\partial P}{\partial z}+\frac{\mu_{hnf}}{\rho_{hnf}}\left(\frac{\partial^{2}w}{\partial r^{2}}+\frac{1}{r}\frac{\partial w}{\partial r}+\frac{\partial^{2}w}{\partial z^{2}}\right),$$

(5) $$u\frac{\partial T}{\partial r}+w\frac{\partial T}{\partial z}=\frac{k_{hnf}}{{\left(\rho c_{p}\right)}_{hnf}}\left(\frac{\partial^{2}T}{\partial r^{2}}+\frac{1}{r}\frac{\partial T}{\partial r}+\frac{\partial^{2}T}{\partial z^{2}}\right)-\frac{1}{{\left(\rho c_{p}\right)}_{hnf}}\frac{\partial q_{r}}{\partial z},$$

along with subject to the boundary conditions

(6) $$\begin{array}{l} \;u=\lambda u_{w}+\frac{2-\sigma_{v}}{\sigma_{v}}\lambda_{0}\frac{\partial u}{\partial z},\;\;v=v_{w},\;\;w=w_{0},\;\;T=T_{w}+\frac{2-\sigma_{T}}{\sigma_{T}}\left(\frac{2\gamma }{\gamma +1}\right)\frac{\lambda_{0}}{\mathrm{Pr}}\frac{\partial T}{\partial z}\;\mathrm{at}\;z=0,\\ \;\;\;\;\;\;\;\;\;\;\;\;\;\frac{\partial u}{\partial z}\to \frac{\partial u_{e}}{\partial z},\;\;v_{e}\to 0,\;\;T\to T_{\infty }\;\;\;as\;\;\;z\to \infty . \end{array}\}$$

In the above governing equations, u, v and w are the component of velocities along r−, θ− and z-axes, P is the pressure, $\sigma_{v}$ is the tangential momentum accommodation coefficient, $\lambda_{0}$ is the coefficient of the main free path, T is the temperature, $\sigma_{T}$ is the thermal accommodation coefficient,γ is the specific heat ratio and λ is the constant stretching/shrinking parameters with λ>0 for the stretching sheet, λ<0 for the shrinking sheet, and λ=0 for the static disk. Further, $\rho_{hnf}$ is the density, $k_{hnf}$ is the thermal conductivity, ${\left(\rho c_{p}\right)}_{hnf}$ is the heat capacity, $\mu_{hnf}$ is the dynamic viscosity, and ${\left(\sigma \right)}_{hnf}$ is the electrical conductivity of the hybrid nanofluid, which is given as (see [19,20]).

(7) $$\left\{ \begin{array}{c} \frac{\mu_{hnf}}{\mu_{f}}=\left(1-\phi \right){\;}^{-2.5}\;\;\;\;where\;\;\;\;\;\phi =\phi_{1}+\phi_{2},\\ \frac{\rho_{hnf}}{\rho_{f}}=\phi_{1}\left(\frac{\rho_{1}}{\rho_{f}}\right)+\phi_{2}\left(\frac{\rho_{2}}{\rho_{f}}\right)+\left(1-\phi \right),\\ \frac{k_{hnf}}{k_{f}}=\left(A_{a}+A_{b}\right)\times {\left(A_{a}+A_{c}\right)}^{-1},\;where\;\;A_{a}=\frac{\left(\phi_{1}k_{1}+\phi_{2}k_{2}\right)}{\phi },\\ A_{b}=2k_{f}+2\left(\phi_{1}k_{1}+\phi_{2}k_{2}\right)-2\phi k_{f},\;A_{c}=2k_{f}-\left(\phi_{1}k_{1}+\phi_{2}k_{2}\right)+\phi k_{f}\\ \frac{{\left(\rho c_{p}\right)}_{hnf}}{{\left(\rho c_{p}\right)}_{f}}=\phi_{1}\left(\frac{{\left(\rho c_{p}\right)}_{1}}{{\left(\rho c_{p}\right)}_{f}}\right)+\phi_{2}\left(\frac{{\left(\rho c_{p}\right)}_{2}}{{\left(\rho c_{p}\right)}_{f}}\right)+\left(1-\phi \right), \end{array}\right.$$

where ϕ corresponding to the solid nanoparticle volume fraction and is equal to the sum of the two distinct nanoparticles such as $\phi =\phi_{1}+\phi_{2}$, in which $\phi_{1}$ signifies the copper (Cu) nanoparticles, $\phi_{2}$ signifies to alumina (Al_2_O_3_) nanoparticles, and ϕ=0 signifies to a regular (viscous) fluid. Besides, $\rho_{f}$,$k_{f}$,${\left(\rho c_{p}\right)}_{f}$, $\rho_{1}$, $\rho_{2}$, $k_{1}$, $k_{2}$,${\left(\rho c_{p}\right)}_{1}$ and ${\left(\rho c_{p}\right)}_{2}$ are the densities, thermal conductivities, and heat capacitance of the (viscous) regular fluid and the hybrid nanoparticles, respectively. Thermophysical properties of the regular (viscous) fluid and both the distinct nanomaterials (copper (Cu) and alumina (Al_2_O_3_)) are written in Table 1.

In energy Equation (5) the last term is used for the radiative heat flux $q_{r}.$ Therefore, the simplified form of the term radiative heat flux using the Rosseland approximation for an optically (hybrid nanoliquid) thick layer one can write (see Hayat et al. [49]):

(8) $$q_{r}=-\frac{4\sigma^{*}}{3k^{*}}\frac{\partial T^{4}}{\partial z},$$

where $\sigma^{*}$ is the Stefan Boltzman constant and $k^{*}$ is the mean absorption constant. Further, executing the well-known Taylor series about the point $T_{\infty }$, the corresponding fourth power of $T^{4}$ can be simplified as $T^{4}≅4T_{\infty }^{3}T-3T_{\infty }^{4}$ by ignoring the higher-order term.

To further, simplify the analysis of the given model, here, we introduce the following self-similarity dimensionless variables are (see Weidman [39]):

(9) $$\begin{array}{l} u\left(r,z\right)=a^{2/3}\upsilon_{f}^{1/3}rF\prime \left(\xi \right),\;v\left(r,z\right)=\omega rG\left(\xi \right),\xi ={\left(a/\upsilon_{f}\right)}^{1/3}z,\\ \theta \left(\xi \right)=\frac{T-T_{\infty }}{T_{w}-T_{\infty }},\;w\left(r,z\right)=-2a^{1/3}\upsilon_{f}^{2/3}F\left(\xi \right), \end{array}$$

where primes correspond the derivative with respect to the pseudo-similarity variable ξ and further the expression (9) leads us to yield:

(10) $$w_{0}=-2a^{1/3}\upsilon_{f}^{2/3}S.$$

In the above Equation (10), the symbol S demonstrates the transparent factor with S>0 and S<0 correspond to suction and blowing, respectively, whereas S=0 is for an impervious surface of the disk.

Now utilizing the self-similarity variables in the governing equations, where Equation (1) is evidently satisfied, while the rest of the Eqs. are transmuted into following ODEs as follows:

(11) $$\frac{\mu_{hnf}/\mu_{f}}{\rho_{hnf}/\rho_{f}}F^{\prime \prime \prime }+2FF^{\prime \prime }-{F^{\prime }}^{2}+\alpha_{A}G^{2}=0,$$

(12) $$\frac{\mu_{hnf}/\mu_{f}}{\rho_{hnf}/\rho_{f}}G^{\prime \prime }+2\left(G^{\prime }F-GF^{\prime }\right)=0,$$

(13) $$\frac{1}{{\left(\rho c_{p}\right)}_{hnf}/{\left(\rho c_{p}\right)}_{f}}\left(k_{hnf}/k_{f}+\frac{4}{3}R_{d}\right)\theta^{\prime \prime }+2\mathrm{Pr}F\theta^{\prime }=0,$$

with appropriate BCs are:

(14) $$\begin{array}{l} F\left(0\right)=S,\;\;\;F^{\prime }\left(0\right)=\lambda +\delta_{1}F^{\prime \prime }\left(0\right),\;\;G\left(0\right)=1,\;\;\theta \left(0\right)=1+\delta_{2}\theta^{\prime }\left(0\right)\\ F^{\prime \prime }\left(\xi \right)\to 2,\;\;G\left(\xi \right)\to 0,\;\;\theta \left(\xi \right)\to 0\;\;\mathrm{as}\;\;\xi \to \infty . \end{array}$$

The above similarity equations comprise some of the dimensionless influential parameters which are namely and symbolically premeditated as like $\mathrm{Pr}=\mu_{f}{\left(c_{p}\right)}_{f}/k_{f}$ the Prandtl number, $\delta_{1}=\left(2-\sigma_{v}/\sigma_{v}\right)\lambda_{0}{\left(a/\upsilon_{f}\right)}^{1/3}$ is the velocity slip parameter, $R_{d}=\left(4\sigma^{*}T_{\infty }^{3}\right)/k_{f}k^{*}$ is the radiation parameter, $\delta_{2}=\left(2-\sigma_{T}/\sigma_{T}\right)\left(2\gamma /\gamma +1\right)\lambda_{0}/\mathrm{Pr}{\left(a/\upsilon_{f}\right)}^{1/3}$ is the temperature slip parameter, and $\alpha_{A}=\omega^{2}/a^{4/3}\upsilon_{f}^{2/3}$ corresponds the rotating disk factor, necessitating that $\alpha_{A}$ is greater or equal to zero.

The important gradients of the problem are the following shear stress in the relative radial and azimuthal directions indicated by $C_{fr}$ and $C_{f\theta }$, respectively, and the local Nusselt number $Nu_{r}$ is defined as

(15) $$\begin{array}{l} C_{fr}=\frac{\mu_{hnf}}{\rho_{f}u_{w}{}^{2}}{\left(\frac{\partial u}{\partial z}\right)|}_{z=0},\;\;C_{f\theta }=\frac{\mu_{hnf}}{\rho_{f}v_{w}{}^{2}}{\left(\frac{\partial v}{\partial z}\right)|}_{z=0},\;\;\\ Nu_{r}=-\frac{r}{k_{f}\left(T_{w}-T_{\infty }\right)}{\left(k_{hnf}\left(\frac{\partial T}{\partial z}\right)-{\left(q_{r}\right)}_{w}\right)|}_{z=0} \end{array}$$

Using similarity transformation (9) in the above equations, we get

(16) Rer1/2Cfr=μhnfμfF″(0), Rer1/2(uwvw)Cfθ=αA−1μhnfμfG′(0), Rer1/2Nur=−(khnfkf+43Rd)θ′(0),

where Rer=uwrυf is called the local Reynolds number.

## 3. Temporal Stability Analysis

The present Agrawal hybrid nanofluid flow problem was solved numerically and admits double solutions (upper branch solution and lower branch solution). Therefore, the multiple branch outcomes for a structure of a different geometry of the problems were documented by Weidman et al. [50], and Chu et al. [51], where the upper branch solution is stable and physically trustworthy, while the lower branch solution is unstable and not physically acceptable. Keeping these published available work in mind, we should test the properties of these stated efforts by letting the two-point problem of Equations (11)–(13). Hence, we define here the new non-dimensional time variable $\Pi =a^{2/3}\upsilon_{f}^{1/3}t$. To test and ease the procedure of the stability analysis, rewrite Equation (9) with new nondimensional variables as follows:

(17) $$\left\{ \begin{array}{l} u=a^{2/3}\upsilon_{f}^{1/3}r\frac{\partial F}{\partial \xi }\left(\xi ,\Pi \right),\;v=\omega rG\left(\xi ,\Pi \right),\xi ={\left(a/\upsilon_{f}\right)}^{1/3}z,\\ \theta \left(\xi ,\Pi \right)=\frac{T-T_{\infty }}{T_{w}-T_{\infty }},\;w=-2a^{1/3}\upsilon_{f}^{2/3}F\left(\xi ,\Pi \right). \end{array}\right.$$

Now executing Equation (17), the governing Equations (11)–(13) along with BCs (14), take the following form:

(18) $$\frac{\mu_{hnf}/\mu_{f}}{\rho_{hnf}/\rho_{f}}\frac{\partial^{3}F}{\partial \xi^{3}}+2F\frac{\partial^{2}F}{\partial \xi^{2}}-{\left(\frac{\partial F}{\partial \xi }\right)}^{2}+\alpha_{A}G^{2}-\frac{\partial^{2}F}{\partial \xi \partial \Pi }=0$$

(19) $$\frac{\mu_{hnf}/\mu_{f}}{\rho_{hnf}/\rho_{f}}\frac{\partial^{2}G}{\partial \xi^{2}}+2\left(\frac{\partial G}{\partial \xi }F-G\frac{\partial F}{\partial \xi }\right)-\frac{\partial G}{\partial \Pi }=0$$

(20) $$\frac{1}{{\left(\rho c_{p}\right)}_{hnf}/{\left(\rho c_{p}\right)}_{f}}\left(k_{hnf}/k_{f}+\frac{4}{3}R_{d}\right)\frac{\partial^{2}\theta }{\partial \xi^{2}}+2\mathrm{Pr}F\frac{\partial \theta }{\partial \xi }-\frac{\partial \theta }{\partial \Pi }=0$$

and the altered BCs become

(21) $$\left\{ \begin{array}{l} \;\frac{\partial F\left(0,\Pi \right)}{\partial \xi }=\lambda +\delta_{1}\frac{\partial^{2}F\left(0,\Pi \right)}{\partial \xi^{2}},\;\;F\left(0,\Pi \right)=S,\;\;G\left(0,\Pi \right)=1,\;\;\theta \left(0,\Pi \right)=1+\delta_{2}\frac{\partial \theta \left(0,\Pi \right)}{\partial \xi }\\ \frac{\partial^{2}F\left(\xi ,\Pi \right)}{\partial \xi^{2}}\to 2,\;\;G\left(\xi ,\Pi \right)\to 0,\;\;\theta \left(\xi ,\Pi \right)\to 0\;\;\mathrm{as}\;\;\xi \to \infty . \end{array}\right.$$

To check the working method of the temporal analysis of the time-independent Agrawal hybrid nanofluid flow outcome $F\left(\xi \right)=F_{0}\left(\xi \right)\;$, $G\left(\xi \right)=G_{0}\left(\xi \right)\;$ and $\theta \left(\xi \right)=\theta_{0}\left(\xi \right)$ satisfy the two-point problem (11) to (13), we write (see Weidman et al. [50], and Chu et al. [51]):

(22) $$F\left(\xi ,\Pi \right)=F_{0}\left(\xi \right)+e^{-\Sigma \Pi }f\left(\xi \right),\;G\left(\xi ,\Pi \right)=G_{0}\left(\xi \right)+e^{-\Sigma \Pi }g\left(\xi \right),\theta \left(\xi ,\Pi \right)=\theta_{0}\left(\xi \right)+e^{-\Sigma \Pi }q\left(\xi \right)$$

Here, the notation Σ is an unknown eigenvalue parameter, and functions f(ξ), g(ξ) and q(ξ) are comparatively small to $F_{0}\left(\xi \right)$,$G_{0}\left(\xi \right)$ and $\theta_{0}\left(\xi \right)$, respectively. Plugging Equation (22) into Equations (18)–(20) along with the boundary conditions (21), the following linear eigenvalue problem is obtained:

(23) $$\frac{\mu_{hnf}/\mu_{f}}{\rho_{hnf}/\rho_{f}}f^{\prime \prime \prime }+2\left(F_{0}f^{\prime \prime }+{F_{0}}^{\prime \prime }f-{F_{0}}^{\prime }f\prime +\alpha_{A}gG_{0}\right)+\Sigma f^{\prime }=0$$

(24) $$\frac{\mu_{hnf}/\mu_{f}}{\rho_{hnf}/\rho_{f}}g^{\prime \prime }+2\left(F_{0}g^{\prime }+{G_{0}}^{\prime }f-G_{0}f^{\prime }-gF_{0}\right)+\Sigma g=0$$

(25) $$\frac{1}{\mathrm{Pr}{\left(\rho c_{p}\right)}_{nf}/{\left(\rho c_{p}\right)}_{f}}\left(k_{nf}/k_{f}+\frac{4}{3}R_{d}\right)q^{\prime \prime }+2\left(F_{0}q\prime +{\theta_{0}}^{\prime }f\right)+\Sigma q=0$$

and the BCs (21) becomes

(26) $$\left\{ \begin{array}{l} \;f^{\prime }\left(0\right)=\delta_{1}f^{\prime \prime }\left(0\right),\;\;f\left(0\right)=0,\;\;g\left(0\right)=0,\;\;q\left(0\right)=\delta_{2}q^{\prime }\left(0\right)\\ f^{\prime \prime }\left(\xi \right)\to 0,\;\;g\left(\xi \right)\to 0,\;\;q\left(\xi \right)\to 0\;\;\mathrm{as}\;\;\xi \to \infty . \end{array}\right.$$

Solving the linear eigenvalue problems (23–25) one observes an infinite number of eigenvalues $\Sigma_{1}<\Sigma_{2}<\Sigma_{3}\ldots$. If we found the Σ (smallest eigenvalue) positive, then the flow is stable and if Σ negative then the flow is unstable.

## 4. Numerical Procedure of the Considered Scheme

In this segment of the paper, we showed the mathematical technique of the studied numerical scheme in comprehensive form, as well as the accuracy of the code for the supplied flow problem. The modified similarity set of ordinary differential Equations (11)–(13) along with the border condition (14) are formed in the extremely nonlinear form, which is quite difficult to solve analytically after applying the similarity variables (9). As a result, these aforementioned numbers of highlighted equations are solved approximately by employing a finite difference scheme, which is known as asbvp4c. This package is a legitimate built-in code for MATLAB software (MATLAB R2020b) that is based on the three-stage Lobatto IIIA formula and was well-described in detail by Shampine et al. [52] and Khan et al. [53]. According to these available articles, the set of higher-order similarity equations were converted into a first-order set of ODEs by incorporating new symbols or notations. To continue our working process of the scheme, let the variables are:

(27) $$F=D_{1},F^{\prime }=D_{2},F^{\prime \prime }=D_{3},G=D_{4},G^{\prime }=D_{5},\theta =D_{6},\theta^{\prime }=D_{7}$$

Now applying the Equation (27) into Equations (11)–(13) with the subject BCs (14), we get the following set of ODEs in the first-order form, which can be written as follows:

(28) $$\frac{d}{d\xi }\left(\begin{array}{l} D_{1}\\ D_{2}\\ D_{3}\\ D_{4}\\ D_{5}\\ D_{6}\\ D_{7} \end{array}\right)=\left(\begin{array}{l} D_{2}\\ D_{3}\\ \frac{\rho_{hnf}/\rho_{f}}{\mu_{hnf}/\mu_{f}}\left(D_{2}{}^{2}-2D_{1}D_{3}-\alpha_{A}D_{4}{}^{2}\right)\\ D_{5}\\ \frac{\rho_{hnf}/\rho_{f}}{\mu_{hnf}/\mu_{f}}\left(2D_{4}D_{2}-2D_{1}D_{5}\right)\\ D_{7}\\ \frac{\mathrm{Pr}{\left(\rho c_{p}\right)}_{hnf}/{\left(\rho c_{p}\right)}_{f}}{\left(k_{hbnf}/k_{bf}+\left(4/3\right)R_{d}\right)}\left(-2D_{1}D_{7}\right) \end{array}\right)$$

with appropriate ICs are

(29) $$\left\{ \begin{array}{l} D_{1}\left(0\right)=S,\;\;\;D_{2}\left(0\right)=\lambda +\delta_{1}D_{3}\left(0\right),\;\;D_{4}(0)=1,\;\;D_{6}\left(0\right)=1+\delta_{1}D_{7}\left(0\right),\\ D_{3}\left(\xi \right)\to 2,\;\;D_{4}\left(\xi \right)\to 0,\;\;D_{6}\left(\xi \right)\to 0\;\;\;\;\;\;\mathrm{at}\;\;\xi \to \infty . \end{array}\right.$$

In ongoing to this process of the problem, we recommended here the essential early estimates at the specific mesh point to grip the system of first-order dimensional forms of Equation (28) with the subjected proper ICs (29), respectively. The mesh size Δξ in ξ, and the thickness of the boundary layer $\xi_{\infty }$ have to be adjusted for dissimilar values of the influential constraints to endure the certain goal of accuracy. This problem has two distinct branch solutions. Therefore, the guess for the solution branch of upper is straightforward, while the solution branch of lower required an appropriate guess, which is quite hard to find out but trying until to satisfy the far-field BCs.

### Validation of the Dual Solutions Code

To check the validation, confirmation or accuracy of the given numerical scheme, a comparison was constructed and shown in this subsection of the work. Table 2 highlights the comparison of wall drag force in the radial direction for several values of $\alpha_{A}$ (without the impact of hybrid nanoparticles, stretching/shrinking parameter, radiation parameter, velocity and temperature slip parameters, and mass flux parameter) with published work of Lok et al. [54]. From this table, we have established that the existing work is completely mapping with exceptional accuracy along with the subject available reported work for the outcome of the first branch solution. Therefore, it revealed an outstanding agreement or sound and give exceptional power to catch or discover the unavailable outcomes using the considered scheme.

## 5. Analysis of Results

**Figure 2:**
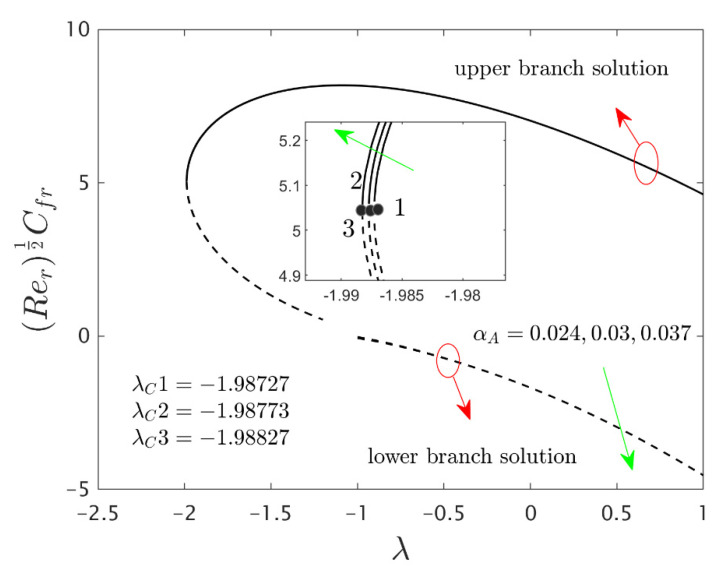
Variations of Rer1/2Cfr against λ for several values of $\alpha_{A}$.

**Figure 3:**
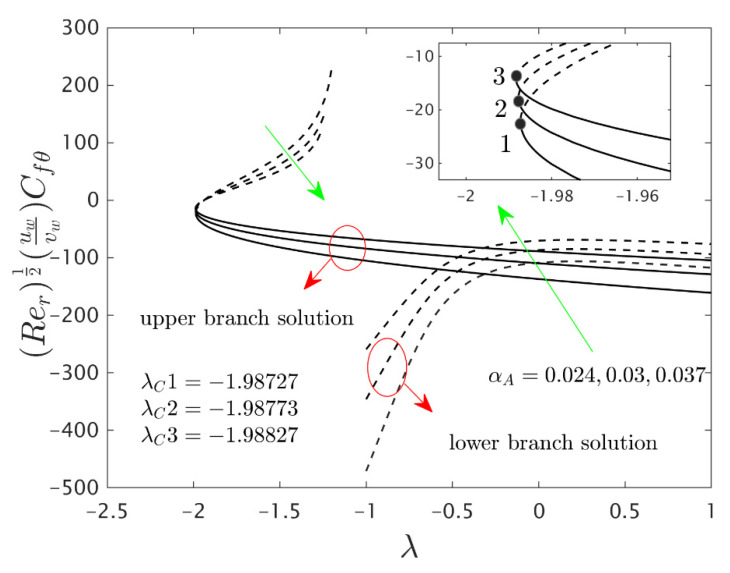
Variations of Rer1/2(uwvw)Cfθ against λ for several values of $\alpha_{A}$.

**Figure 4:**
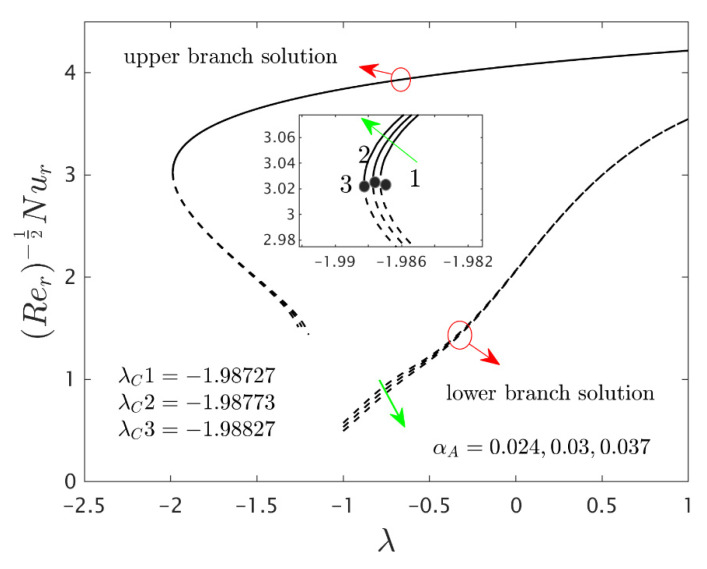
Variations of Rer−1/2Nur against λ for several values of $\alpha_{A}$.

**Figure 5:**
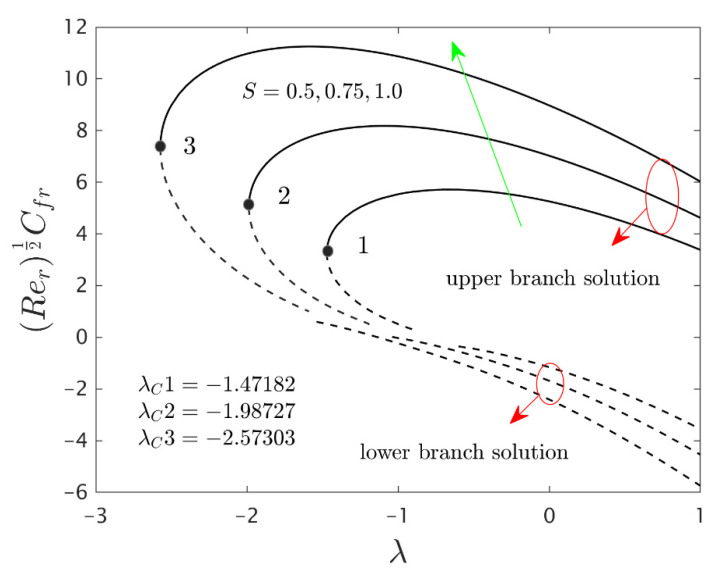
Variations of Rer1/2Cfr against λ for several values of S.

**Figure 6:**
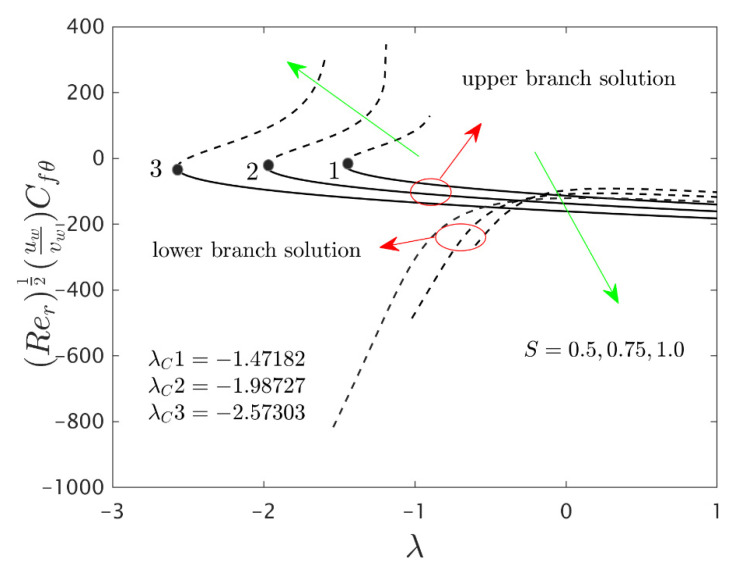
Variations of Rer1/2(uwvw)Cfθ against λ for several values of S.

**Figure 7:**
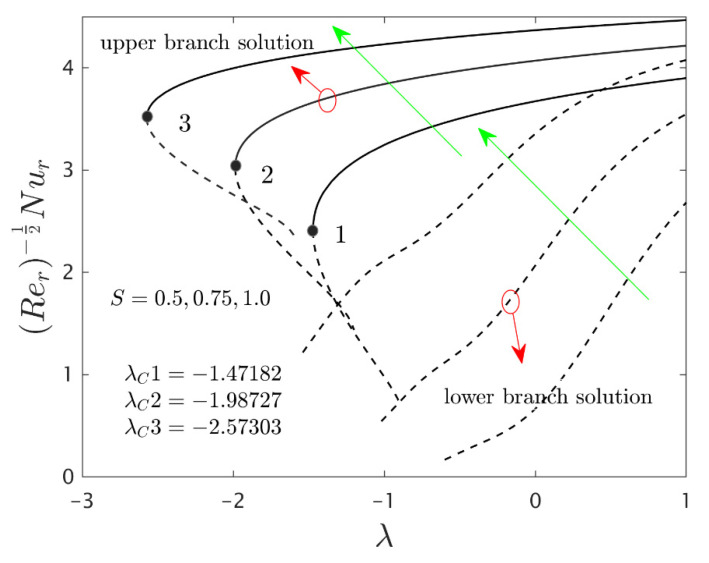
Variations of Rer−1/2Nur against λ for several values of S.

**Figure 8:**
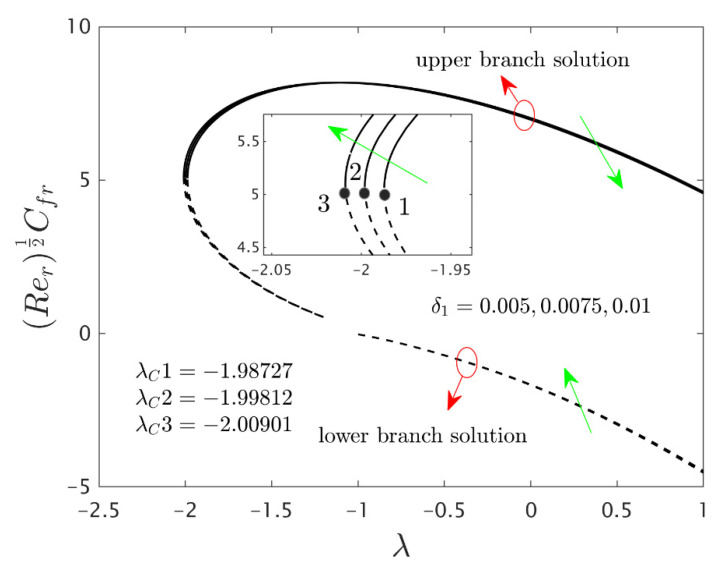
Variations of Rer1/2Cfr against λ for several values of velocity slip parameter.

**Figure 9:**
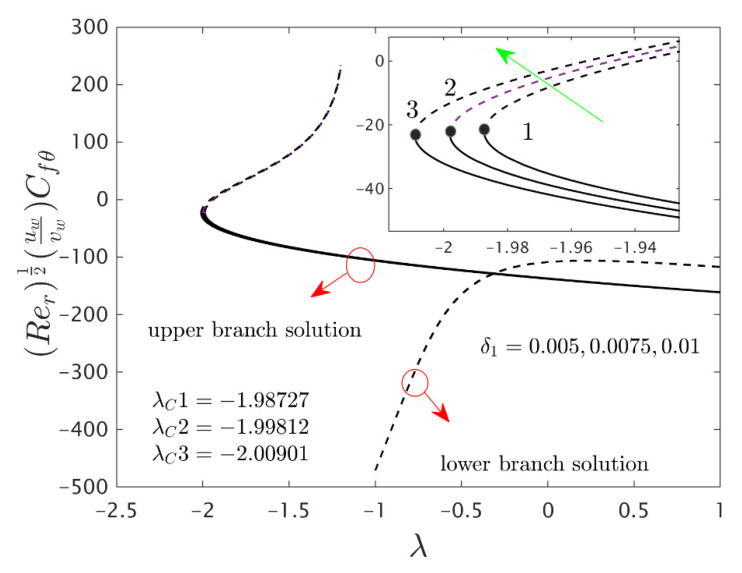
Variations of Rer1/2(uwvw)Cfθ against λ for several values of velocity slip parameter.

**Figure 10:**
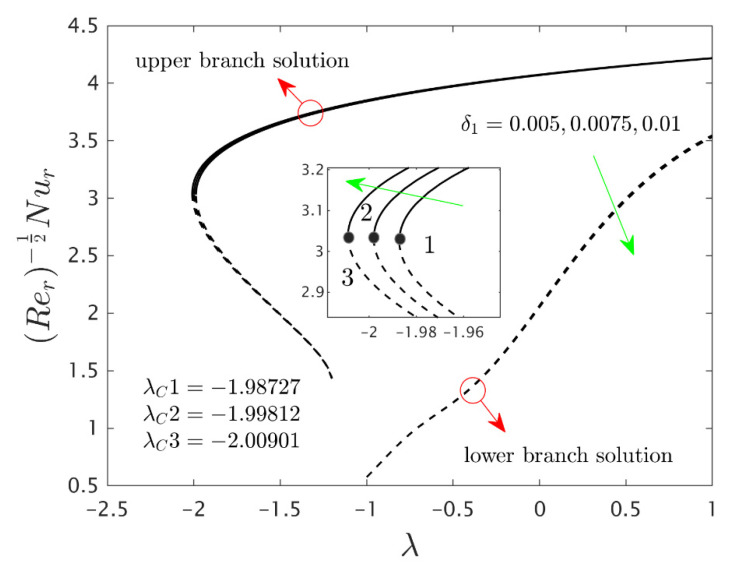
Variations of Rer−1/2Nur against λ for several values of velocity slip parameter.

**Figure 11:**
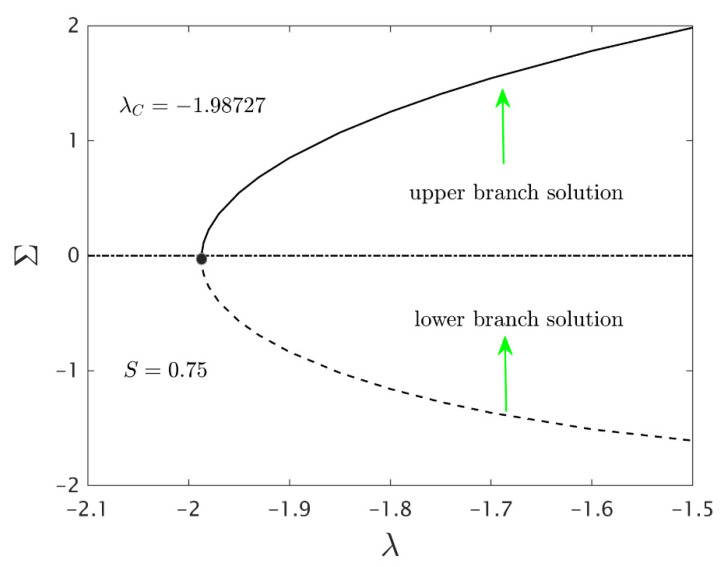
Smallest eigenvalues Σ for different values of λ.

The similarity equations contained distinct controlling parameters like radiation parameter $R_{d}$, rotating disk parameter $\alpha_{A}$, velocity slip parameter $\delta_{1}$, stretching/shrinking parameter λ, mass suction parameter S, temperature slip parameter $\delta_{2}$, and the hybrid nanoparticles $\phi_{1}$ and $\phi_{2}$. For the persistence of the code simulations, we guaranteed the following range values of the selected distinguished constraints like $0.0<R_{d}<5.0$, $0.0<\alpha_{A}\leq 0.5$, $0.01<\delta_{1}<2.0$, $0.0<\delta_{2}<2.0$, 0.0<S<5.0, −5.0<λ<2.0,$0.02<\phi_{1}<0.04$ and $0.02<\phi_{2}<0.04$, whereas, the Prandtl number Pr=6.2. Furthermore, the consequence of these selected comprised parameters on the wall drag forces along the radial and azimuthal directions, eigenvalues, and heat transfer of the hybrid (Cu-Al_2_O_3_/water) nanoparticles for the two distinct (first and second) branch solutionsversus λ are portrayed in Figure 2, Figure 3, Figure 4, Figure 5, Figure 6, Figure 7, Figure 8, Figure 9, Figure 10 and Figure 11, respectively, whereas the Table 3 and Table 4 are also prepared for the computational values of the gradients for several distinct values of the pertinent included influential parameters. More accurately, the numerical values of the wall drag forces along the radial and azimuthal directions for the sundry values of $\alpha_{A}$, S, $\delta_{1}$, $\phi_{1}$ and $\phi_{2}$ are constructed in Table 3 when λ=−1.4 (shrinking sheet), $R_{d}=1.5$ and $\delta_{2}=0.5$. Meanwhile, Table 4 exhibitions the heat transfer numerical values for the enormous distinct varying values of $\alpha_{A}$, S, $\delta_{1}$,$R_{d}$,$\delta_{2}$, $\phi_{1}$ and $\phi_{2}$ when λ=−1.4 (shrinking sheet)and Pr=6.2. Table 5, Table 6 and Table 7 are prepared to compute values of normal nanofluid, and pure fluid as well as the presence and absence of slip factors. From the outcome of the tables, it is observed that the friction factor in the radial direction and heat transfer enriches for the first and second branch solutions with higher values of $\alpha_{A}$,S, $\phi_{1}$ and $\phi_{2}$. Meanwhile, the friction factor reduces in both branches with higher values of S but it is decreases and increases with hybrid nanoparticles and then increases and reduces in the first and second branch solution with $\alpha_{A}$. Moreover, the wall drag force in the radial direction and heat transfer rises for the first branch and decline for the second branch with larger values of $\delta_{2}$ whereas the shear stress along the azimuthal direction abruptly shrinkages for both branches. Besides, the heat transfer decays and develops for the first and second branch outcomes with superior values of $\delta_{2}$ and $R_{d}$, respectively. Physically, the fluid particles captivate additional heat transfer by booming the parameter $R_{d}$ as a consequence, the Nusselt number upsurges. Moreover, the shear stress and heat transfer rate uplift as the velocity slip as well as temperature slip parameters enhance. Furthermore, with the presence of hybrid nanofluids, the heat transfer enhances further. Therefore, the performance of the cooling will be efficient for the inclusion of hybrid nanoparticles. Furthermore, the heat transfers and the magnitude of the friction factors in the radial and azimuthal directions in the stable branch are higher for the nanofluid as compared to the working base fluid.

The deviations of the rotating disk parameter $\alpha_{A}$ on the wall drag forces along the radial and the azimuthal directions and heat transfer $-\left(k_{hnf}/k_{f}+\left(4/3\right)R_{d}\right)\theta ’\left(0\right)$ of the hybrid (Cu-Al_2_O_3_/water) nanoparticles for the two distinct (first and second) branch solutions against λ are bounded in Figure 2, Figure 3 and Figure 4, respectively. From the graphs, we have observed that the wall drag force in the radial direction and rate of heat transfer upsurge for the first outcome and reduces for the second outcome with improving values of the parameter $\alpha_{A}$, while the trend of the shear stress in the azimuthal direction continuously progresses for both distinct branch outcomes. Physically, the deceleration of velocity in the azimuthal direction is due to the fact of higher values of the rotating disk parameter which gets moderately transmitted to the upcoming adjacent fluid layers. As a result, the wall drag force in the azimuthal direction elevates. The branch of first outcomes is continuously smooth and no breaking exists for higher $\alpha_{A}$ (see Figure 2, Figure 3 and Figure 4) while the line was breaking at some finite values of $\lambda_{0}$ of λ due to the rotating disk parameter. Further, the first and second branch outcome was constructed in these graphs owing to larger $\alpha_{A}$ for the phenomena of stretching and shrinking parameter. In other words, the nonunique outcomes were possible to find for the case of λ<0 and λ>0, whereas the outcome was unique for $\lambda =\lambda_{C}$ and no solution was accessible to found for $\lambda <\lambda_{C}$. For higher values of $\alpha_{A}$, the following three distinct critical points are obtained and were written in each window of Figure 2, Figure 3 and Figure 4, respectively. These points are also emphasized in the zoom window of the pictures by a sequence number such as 1, 2, and 3, while it is also spotted by the solid black balls. For mounting values of $\alpha_{A}$, the absolute values of the bifurcation points elevate, which shows that the trend of the separation of the boundary layer declines.

Figure 5, Figure 6 and Figure 7 elucidate the influence of S on the wall drag forces along the radial and azimuthal directions and heat transport of the hybrid nanomaterials for the two individual (first and second) branch solutions against λ, respectively. The wall drag force in the radial direction enlarges for the first outcome and declines in the unstable outcome owing to the higher impacts of S. Physically, an improvement in S conveys the flow of hybrid nanoparticles near the surface of the rotating disk which can bring velocity profile down, and consequently, the drag coefficient along with the radial direction upsurges. On the other hand, the $-\left(k_{hnf}/k_{f}+\left(4/3\right)R_{d}\right)\theta ’\left(0\right)$ develops in both solution branches for the higher consequences of the parameter S while the wall drag force along with the azimuthal directions abruptly declines for both branches and escalates near the bifurcation points for the unstable branch outcomes with S. Moreover, the second branch results of the key physical quantities of interest are breaking or disconnected through some finite values of $\lambda_{0}$ of λ when the values of S is increasing. The gap in the first solution curves is finer as related to the distance in the second solution curves for the distinct selected values of S. With the variant values of S, we have perceived the following bifurcation values −1.47182, −1.98727 and −2.57303, respectively. From these critical points, it is seen that higher values of S the solution domain expands faster towards the larger negative values of λ and therefore, the magnitude of the bifurcation values is also boosted up.

Furthermore, the power of the velocity slip parameter $\delta_{1}$ on the wall drag forces along the radial and azimuthal directions and heat transfer of the hybrid nanoparticles are depicted in Figure 8, Figure 9 and Figure 10, respectively. The wall drag force in the radial direction and heat transfer enrich for the first solution and moderates for the second outcome due to the augmentation $\delta_{1}$, while the trend of the wall drag force in the radial direction is completely changed only in the case of stretching for both branches with higher values of $\delta_{1}$ (see Figure 8). Generally, in the phenomenon of Maxwell slip BCs, the speed of the disk and the fluid particles are not the same at the disk surface which depreciates the fluid speed and generates a reduction in the velocity profile as a conclusion, the wall drag force in the relevant radial direction escalates. Alternatively, the wall drag force along the azimuthal direction decelerates for both branches of outcome with superior impacts of $\delta_{1}$ while the tendency or behavior of the flow near the bifurcation values for the second branch rises owing to the increment in $\delta_{1}$. In addition, the impacts of the velocity slip parameter $\delta_{1}$ on the gradients are very small, and therefore we can zoom a specific part of the graphs to show the difference between the first curves and second curves. The zooming portion of the graphs also comprised the associated bifurcation values for the corresponding values of $\delta_{1}$, which can also highlight in these figures by positions 1, 2, and 3 respectively. Similarly, here the second branch outcome breaks at some specific point against the stretching/shrinking parameter for the larger values of $\delta_{1}$ due to the non-rotating disk parameter.

Finally, Figure 11 exposes the smallest eigenvalues of Σ for the numerous values of the stretchable/shrinkable parameter λ. From the constructed graph, the negative value of Σ specifies an initial growth of disturbance, and the flow is an unstable mode. On the other hand, the flow is said to be in a stable mode when the value of Σ are positive which creates the initial decay of disturbance. In addition, we can noticed that the value of Σ tends to zero either from the lower branch or the upper branch solution as the values of λ are approaching, $\lambda_{C}$. This shows that the transitions from positive (stable) to negative (unstable) of Σ occur at the turning points.

## 6. Conclusions

In this idea of the manuscript, we studied the impact of Smoluchowski temperature and Maxwell velocity slip boundary conditions on thermal radiative axisymmetric rotational stagnation-point flow induced by hybrid (Cu-Al_2_O_3_/water) nanoparticles impinging on a porous stretchable/shrinkable rotating disk with heat transfer analysis. The similarity Equations (11)–(13) along with BCs (14) were reduced from the governing PDEs via exercising the pertinent self-similarity variables. The obtained set of similarity equations comprised distinct dimensionless controlling parameters. Therefore, the multiple outcomes for various involved influential parameters on engineering physical quantities of interest for the first and second branch outcomes were demonstrated through various dissimilar graphs and as well as in a tabular form. The main findings of the considered simulations are summarized as follows:

❖ The wall drag force coefficient in the radial direction and heat transfer upsurges for the branch of the first outcome and reduces for the branch of the second outcome owing to higher values of $\alpha_{A}$. However, the shear stress along the azimuthal direction elevates for both branches with $\alpha_{A}$, while the trends or behaviors are inverted near the bifurcation values for the branch of second solution curves.
❖ Improvement in the mass suction parameter S displays an enhancement in the heat transfer, but the reduction in the shear stress along the azimuthal direction for both solution branches while the wall drag force in the radial direction rises for the first branch and declines for the second branch curves.
❖ The heat transfer develops with radiation parameter for both solution branches, while shrinkages due to the temperature slip parameter $\delta_{2}$.
❖ The shear stress along the radial direction upturns for the first solution and drops for the second solution with an enhancement in $\delta_{1}$, while the wall drag force in the azimuthal direction diminished for both solution branches, but heat transfer upsurges for the first branch and decays for the second branch with higher values of $\delta_{1}$.
❖ An improvement in the heat transfer is also remarked with the larger impacts of hybrid nanoparticles. On the other hand, the shear stress in the radial direction augments for the first branch and reduces for the second branch with a development in the hybrid nanoparticles, but the impact of the parameter is contrary for the shear stress in the azimuthal direction.
❖ Due to the temporal stability analysis, the lower branch solution is unstable while the upper branch solution is physically stable.

## Figures and Tables

**Table 1:** Thermophysical properties of regular fluid and hybrid nanoparticles [43].

Properties	$\rho \left(kg/m^{3}\right)$	$c_{p}\;\left(J/KgK\right)$	k (W/mK)	Pr
Water	997.1	4179	0.613	6.2
Cu	8933	385	400	-
Al_2_O_3_	3970	765	40	-

**Table 2:** Comparison of $F^{\prime \prime }(0)$ with Lok et al. [54] for different values of $\alpha_{A}$ when $\phi_{1}=\phi_{2}=S=\delta_{1}=\delta_{2}=0$, and λ=0.

$\alpha_{A}$	Present Results	Lok et al. [54]
0.0	2.0000000	2.00000
25.0	6.8191539	6.81915
100.0	17.055614	17.05561
225.0	30.488228	30.48822
400.0	46.443400	46.44340
625.0	64.560920	64.56092
900.0	84.604186	84.60416
∞	-	-

**Table 3:** Values of shear stress along radial and azimuthal directions for several values of selected parameters when λ=−1.4 (shrinking sheet), $R_{d}=1.5$ and $\delta_{2}=0.5$.

$\phi_{1},\phi_{2}$	S	$\alpha_{A}$	$\delta_{1}$	$\frac{\mu_{hnf}}{\mu_{f}}F^{\prime \prime }\left(0\right)$		$\alpha_{A}{}^{-1}\frac{\mu_{hnf}}{\mu_{f}}G^{\prime }\left(0\right)$	
				Upper Solution	Lower Solution	Upper Solution	Lower Solution
0.024	0.5	0.025	0.05	5.2107	0.4136	−59.1003	619.7336
0.025				5.2508	0.4187	−59.6113	623.8548
0.026				5.2908	0.4237	−60.1223	627.9888
0.024	0.4	0.025	0.05	3.1207	0.1921	−23.5746	629.9871
	0.45			4.4412	0.3076	−47.2809	625.8405
	0.5			5.2107	0.4136	−59.1003	619.7336
0.024	0.5	0.025	0.05	5.2107	0.4136	−59.1003	619.7336
		0.030		5.2131	0.4466	−49.2660	466.9891
		0.035		5.2154	0.4764	−42.2415	367.3144
0.024	0.5	0.025	0.01	4.5402	0.4132	−42.2035	630.2588
			0.05	5.2107	0.4136	−59.1003	619.7336
			0.09	5.5504	1.4286	−71.7705	28.1480

**Table 4:** Values of heat transfer rate for several values of selected parameters when λ=−1.4 (shrinking sheet) and Pr=6.2.

$\phi_{1},\phi_{2}$	S	$\alpha_{A}$	$\delta_{2}$	$R_{d}$	$\delta_{1}$	$-\left(\frac{k_{hnf}}{k_{f}}+\frac{4}{3}R_{d}\right)\theta^{\prime }\left(0\right)$	
						Upper Solution	Lower Solution
0.024	0.5	0.025	0.5	1.5	0.05	3.1184	0.0491
0.025						3.1238	0.0508
0.026						3.1292	0.0524
0.025	0.4	0.025	0.5	1.5	0.05	2.3547	0.0091
	0.45					2.8656	0.0220
	0.5					3.1184	0.0491
0.024	0.5	0.025	0.5	1.5	0.05	3.1184	0.0491
		0.030				3.1186	0.0551
		0.035				3.1189	0.0611
0.024	0.5	0.025	0.5	1.5	0.05	3.1184	0.0491
			0.6			2.8374	0.0490
			0.7			2.6028	0.0489
0.025	0.5	0.025	0.5	1.5	0.05	3.1184	0.0491
				2.0		3.5381	0.1147
				2.5		3.9345	0.2087
0.025	0.5	0.025	0.5	1.5	0.01	2.8740	0.0458
					0.05	3.1184	0.0491
					0.09	3.2840	1.6669

**Table 5:** Values of shear stress along radial direction for several values of selected parameters when λ=−1.1 (shrinking sheet), $\delta_{1}=0$, $\phi_{2}=0$, $\delta_{2}=0$ and $R_{d}=1.5$.

S	$\alpha_{A}$	$\frac{\mu_{hnf}}{\mu_{f}}F^{\prime \prime }\left(0\right)\left(\phi_{1}=0.024\right)$		$\frac{\mu_{hnf}}{\mu_{f}}F^{\prime \prime }\left(0\right)\left(\phi_{1}=0.0\right)$	
		Upper Solution	Lower Solution	Upper Solution	Lower Solution
0.35	0.025	3.0531	1.1752	2.8623	−0.5605
0.40	-	3.6671	0.9540	3.4362	−0.4564
0.45	-	4.2147	0.8049	3.9479	−0.3589
0.40	0.025	3.6671	0.9540	3.4362	−0.4564
-	0.030	3.6695	0.9529	3.4384	−0.4923
-	0.035	3.6719	0.2255	3.4407	−0.5260

**Table 6:** Values of shear stress along azimuthal direction for several values of selected parameters when λ=−1.1 (shrinking sheet), $\delta_{1}=0$, $\phi_{2}=0$, $\delta_{2}=0$ and $R_{d}=1.5$.

S	$\alpha_{A}$	$\alpha_{A}{}^{-1}\frac{\mu_{hnf}}{\mu_{f}}G^{\prime }\left(0\right)\left(\phi_{1}=0.024\right)$		$\alpha_{A}{}^{-1}\frac{\mu_{hnf}}{\mu_{f}}G^{\prime }\left(0\right)\left(\phi_{1}=0.0\right)$	
		Upper Solution	Lower Solution	Upper Solution	Lower Solution
0.35	0.025	−31.7047	12.7473	−29.6897	−431.9075
0.40	-	−41.0618	24.5273	−38.4247	−437.2524
0.45	-	−48.6870	36.2003	−45.5458	−441.5778
0.40	0.025	−41.0618	24.5273	−38.4247	−437.2524
-	0.030	−34.2330	20.6380	−32.0345	−335.3745
-	0.035	−29.3552	16.7707	−27.4700	−268.1761

**Table 7:** Values of heat transfer rate for several values of selected parameters when λ=−1.1 (shrinking sheet), Pr=6.2, $\delta_{1}=0$, $\delta_{2}=0$ and $\phi_{2}=0$.

S	$\alpha_{A}$	$R_{d}$	$-\left(\frac{k_{hnf}}{k_{f}}+\frac{4}{3}R_{d}\right)\theta^{\prime }\left(0\right)\left(\phi_{1}=0.024\right)$		$-\left(\frac{k_{hnf}}{k_{f}}+\frac{4}{3}R_{d}\right)\theta^{\prime }\left(0\right)\left(\phi_{1}=0.0\right)\;9.9077\times 10^{-5}\;4.0460\times 10^{-4}\;4.0460\times 10^{-4}\;3.1873\times 10^{-4}\;2.5452\times 10^{-4}\;4.0460\times 10^{-4}$	
			Upper Solution	Lower Solution	Upper Solution	Lower Solution
0.35	0.025	1.5	3.9657	1.6691	3.9295	$9.9077\times 10^{-5}$
0.40	-	-	4.8073	1.5008	4.7742	$4.0460\times 10^{-4}$
0.45	-	-	5.5618	1.4256	5.5323	0.0014
0.40	0.025	1.5	4.8073	1.5008	4.7742	$4.0460\times 10^{-4}$
-	0.30	-	4.8084	1.4925	4.7752	$3.1873\times 10^{-4}$
-	0.35	-	4.8095	0.0306	4.7763	$2.5452\times 10^{-4}$
0.40	0.025	1.5	4.8073	1.5008	4.7742	$4.0460\times 10^{-4}$
-	-	2.0	5.2159	1.7736	5.1827	0.0028
-	-	2.5	5.6173	2.0387	5.5847	0.0105

## Data Availability

Not applicable.

## Nomenclature

(r,θ,z)	Cylindrical coordinate system (m)
$w_{0}$	Constant mass flux velocity (m/s)
$T_{\infty }$	Constant ambient temperature (K)
P	Pressure (Kg/m. s^2^)
k	Thermal conductivity (W/(m. K))
$q_{r}$	Radiative heat flux
Pr	Prandtl number
T	Temperature of the fluid (K)
$c_{p}$	Specific heat at constant pressure (J/Kg. K)
F(ξ),G(ξ)	Dimensionless velocity stream function
$T_{w}$	Constant surface temperature (K)
S	Mass suction parameter
$v_{w}$	Rotating velocity of the disk (m/s)
$R_{d}$	Radiation parameter
$u_{w}$	Surface velocity of the disk (m/s)
$Nu_{r}$	Local Nusselt number
$u_{e},v_{e},w_{e}$	Free-stream velocities (m/s)
$C_{fr}$	Skin friction coefficient along the radial direction
a	Constant parameter having units (m. s)^−1^
$C_{f\theta }$	Skin friction coefficient along the azimuthal direction
(u,v,w)	Velocity components (m/s)
Rer	Local Reynolds number
***Greek Symbols***	
α	Thermal diffusivity (m^2^/s)
$\upsilon_{f}$	Kinematic viscosity (m^2^/s)
$\alpha_{A}$	Rotating disk parameter
$\sigma_{v}$	Tangential momentum accommodation coefficient
$\lambda_{0}$	Coefficient of the main free path
θ(ξ)	Dimensionless temperature
$\sigma_{T}$	Thermal accommodation coefficient
ω	Constant angular velocity (m/s)
γ	Specific heat ratio
λ	Stretching/Shrinking parameter
$\sigma^{*}$	Stefan-Boltzmann constant (W/(m^2^. K^4^))
μ	Absolute viscosity (N. s/m^2^)
ξ	Pseudo-similarity variable
ρ	Density (kg/m^3^)
$k^{*}$	Mean absorption constant
ψ	Stream function
$\delta_{1}$	Velocity slip parameter
ϕ	Solid volume fraction of nanoparticles
$\delta_{2}$	Temperature slip parameter
***Acronyms***	
Cu	Copper
PDEs	Partial differential equations
H_2_O	Water
ODEs	Ordinary differential equations
bvp4c	Boundary value problem of the fourth order
Al_2_O_3_	Alumina
2D, 3D	Two and three-dimensional
BCs	Boundary conditions
N-S	Navier-Stokes equation
ICs	Initial conditions
***Subscripts***	
hnf	Hybrid nanofluid
1,2	Hybrid nanoparticles (Cu and Al_2_O_3_)
f	Working base fluid
w	Wall boundary condition
∞	Far-field condition
***Superscript***	
*′*	Derivative with respect to ξ
